# The ‘Ethical’ COVID-19 Vaccine is the One that Preserves Lives: Religious and Moral Beliefs on the COVID-19 Vaccine

**DOI:** 10.1093/phe/phab018

**Published:** 2021-07-19

**Authors:** Alberto Giubilini, Francesca Minerva, Udo Schuklenk, Julian Savulescu

**Affiliations:** 2 Department of Philosophy, University of Milan; 3 Queen’s University; 4 Oxford Uehiro Centre for Practical Ethics, University of Oxford; Visiting Professorial Fellow in Biomedical Ethics, Murdoch Childrens Research Institute; Distinguished Visiting International Professorship in Law, University of Melbourne

## Abstract

Although the COVID-19 pandemic is a serious public health and economic emergency, and although effective vaccines are the best weapon we have against it, there are groups and individuals who oppose certain kinds of vaccines because of personal moral or religious reasons. The most widely discussed case has been that of certain religious groups that oppose research on COVID-19 vaccines that use cell lines linked to abortions and that object to receiving those vaccine because of their moral opposition to abortion. However, moral opposition to COVID-19 vaccine research can be based on other considerations, both secular and religious. We argue that religious or personal moral objections to vaccine research are unethical and irresponsible, and in an important sense often irrational. They are unethical because of the risk of causing serious harm to other people for no valid reason; irresponsible because they run counter to individual and collective responsibilities to contribute to important public health goals; and in the case of certain kinds of religious opposition, they might be irrational because they are internally inconsistent. All in all, our argument translates into the rather uncontroversial claim that we should prioritize people’s lives over religious freedom in vaccine research and vaccination roll out.

## Introduction

The COVID-19 pandemic should not have been an unexpected event, since pandemics or large epidemics periodically occurred throughout human history (Garrett, 1994). Indeed, new pandemics are likely to occur again and to be more virulent than this one. The ‘Spanish Flu’ pandemic in 1918 killed at least 50 million people and infected 500 million people worldwide. The ‘Black Death’ plague ‘devastated the Western world from 1347 to 1351, killing 25–50% of Europe’s population and causing or accelerating marked political, economic, social and cultural changes’ (Gottfried, 1983) Around 75 million people perished at the time (Sandle, 2013). By October 2020, COVID-19 has reportedly killed directly over 1 million people all over the world in slightly less than a year (Musil, 2020). 

To prevent or contain such outbreaks today, it is necessary to not only develop safe and effective vaccines but to also do it as quickly as possible and to make them accessible to as many people as possible. This requires pursuing more lines of research at the same time, given that inevitably many of them will be unsuccessful or would require longer than others to be completed. It is also important to have different types of vaccines in order to maximize the chances of conferring protection against new variants of the virus and of conferring adequate protection to different population groups. The longer we go without adequate vaccine supply, the more people will die of COVID-19 (or other viruses that might appear in the future) or of the consequences of pandemic management measures [such as declaring cancer care non-urgent, resulting in a spike of people presenting at hospitals with advanced cancer (Ogilvie, 2020)]. However, the urgency of developing vaccines does not mean that research, development, distribution and vaccination policies may bypass ethical and scientific standards (London and Kimmelman, 2020). ‘As quickly as possible’ means as quickly as reasonable ethical and scientific standards allow.

What constitutes an ethical vaccine research and policy has recently been the subject of some controversy involving religious authorities and scholars defending religious freedom, particularly those from the Christian tradition. Religious objections are not the only type of moral objections to certain vaccines. The arguments we are going to provide in this paper apply to a number of religious and non-religious positions on vaccine research alike. Actually, as we shall see, the fact that there are many possible kinds of moral objections to certain kinds of vaccine research is itself a further reason in support of our line of argument, as it makes respect for freedom of conscience with regard to vaccine choice even more difficult to justify. However, we are going to focus primarily on religious objections to vaccine research that uses foetal cell lines because it has been more prominent in the debate and because it could potentially influence the vaccination choice of a very large number of people.

Ethical considerations, when sound, can legitimately slow down vaccine development and uptake. Because slowing down vaccine development and uptake likely results in avoidable deaths, it is important to assess whether these ethical considerations are legitimate and what ethical weight they should be given, if any. At the moment, it is difficult to estimate or predict how many people have refused or will refuse a certain vaccine because of these kinds of personal ethical concerns. The rate may be very different in different countries or cultural contexts. But the number is potentially very high at least in certain contexts, given the number of ethical issues that vaccine development raises and the number of followers of certain religions.

In particular, two types of concerns have been raised: about vaccine research itself and about future vaccination policies.

With regard to the first, a lot of publicity, including by highly respected scientific journals (Wadman, 2020a), has been given to views expressed by some Catholic, Anglican and Greek Orthodox authorities in the USA, Canada and Australia. They have condemned research on the COVID-19 vaccine that uses cell lines obtained from tissues harvested from aborted foetuses. (Henceforth, we will refer to such vaccines as ‘vaccines linked to abortion’.)

Why are human foetal cell lines being used in research? One major approach to developing a new vaccine for corona virus is to use viral vectors, such as adenovirus. Johnson and Johnson in the USA and the University of Oxford (partnered with the pharmaceutical company AstraZeneca) have used adenovirus approaches. These require the use of cell lines such as the HEK (human embryonic kidney) 293 cell-line, which is derived from tissues of a foetus aborted in 1972 (Wadman, 2020b).

HEK293 cell lines enable the deletion of genes so that the adenovirus vector does not replicate in the vaccinee’s cells (He *et al*., 1998; Thomas and Smart, 2005). Thus, the virus contains the gene which stimulates production of COVID-19 proteins, which elicit immunity, without overwhelming cells with the virus, which can happen with natural infection. In other words, HEK293 cell lines make vaccine development efficient, because they contain the genetic material necessary to produce the desired adenoviruses and that cannot be obtained through animal cell lines, and safe, because the resulting adenovirus would not contain the genes that would replicate the virus and infect the cells of the vaccinees.

The Pfizer/BioNTech vaccine does not use foetal cell lines in the development of the vaccine, although it did use it to test the vaccine. For those who object to research linked to abortion, it might not be ‘ethically uncontroversial’, as some have called it (e.g. Sherley and Prentice, 2020), but to many, it is less controversial than, for example, the Oxford/AstraZeneca vaccine. It might be objected that this makes it unnecessary to use vaccines using an adenovirus. But this would be a mistake. We need as many different vaccines as possible. Besides, there are serious drawbacks with an mRNA vaccine. It must be stored at −70°C (−94 F) and it can’t be removed from the fridge more than four times.^1^ This severely limits its distribution, especially considering that it must be administered in two doses. Many countries have already invested and signed agreements for the production of adenovirus vaccines. For quite some time, there will be vaccine shortages and because in a pandemic time is lives, changing vaccine may cost significant numbers of lives. The more vaccines are approved, the more availability we will have in the shorter term, and the more lives will be preserved.

So how should we evaluate ethical opposition to the use of vaccines which use aborted foetal material in their production?

The Congregation for the Doctrine of Faith, which is taken by the Roman Catholic Church as source of authoritative teaching, has stated that use of already existing COVID-19 vaccines derived from tissues harvested from foetuses after elective abortions can be morally permissible (Congregation for the Doctrine of Faith, 2020). However, this consideration is not taken to apply to *novel* research on vaccines. In a letter addressed to the US Food and Drug Administration, and signed by a number of American Bishops, Archbishops, the President of the Catholic Medical Association and a group representing socially conservative paediatricians, the signatories.

‘strongly urge [the] federal government to ensure that fundamental moral principles are followed in the development of such vaccines, most importantly, the principle that human life is sacred and should never be exploited, [and] urgently and respectfully implore [the US Government] to not only ensure that Americans will have access to a COVID vaccine that is free of ethical concerns, but to encourage and incentivize pharmaceutical companies to use only ethical cell lines or processes for producing vaccines’.^2^

A second concern is that governments should ensure that people are left with alternatives to what they consider an unethical vaccine. If the state cannot or does not want to guarantee that an alternative vaccine is available by allocating research funding accordingly, then, they argue, a moral conflict can arise between the duty to get vaccinated and the duty not to use unethical vaccines. For instance, according to the Catholic Archbishop of Sydney Anthony Fisher, ‘those who are troubled by [the COVID-19 vaccine] will either have to acquiesce to the social pressure to use the vaccine on themselves and their dependents, or conscientiously object to it’.^3^

It is worth stressing that the Congregation for the Doctrine of Faith has issued a specific document stating the moral permissibility for Catholics to use COVID-19 vaccines linked to abortion in the current situation. According to the note, ‘all vaccinations recognized as clinically safe and effective can be used in good conscience with *the certain knowledge that the use of such vaccines does not constitute formal cooperation with the abortion* from which the cells used in production of the vaccines derive’ (Congregation for the Doctrine of Faith, 2020).

However, the Congregation is also very clear that this consideration only applies if alternative, more ethical vaccines are not available, ‘(e.g. in countries where vaccines without ethical problems are not made available to physicians and patients, or where their distribution is more difficult due to special storage and transport conditions, or when various types of vaccines are distributed in the same country but health authorities do not allow citizens to choose the vaccine with which to be inoculated)’ (Congregation for the Doctrine of Faith, 2020). Besides, freedom of conscience and religious freedom are not conditional upon adherence to some official doctrine. Part of the notion of religious freedom is the freedom to choose which religious authorities to follow and the dictates of one’s own conscience (thus, for instance, a Catholic in Australia might want to follow the recommendation of the local Archbishop rather than of the Roman authority).

The issue of conscientious objection to vaccination—that is, the refusal to comply with certain vaccination requirements because of personal moral or religious views (as opposed to refusal motivated by concerns around safety or effectiveness of vaccines)—has been discussed in recent years (e.g. Clarke *et al*., 2017; Giubilini *et al*., 2017; Navin and Largent, 2017; Giubilini, 2019). Some of those who have previously defended such a right in the name of religious freedom or freedom of conscience (e.g. Navin and Largent, 2017) have endorsed the stance of these religious leaders with regard to the future COVID-19 vaccine (Navin and Redinger, 2020). The general principle regulating conscientious objection to vaccination, according to Mark Navin and Mark Largent, is taken to be the following:

‘it is morally justifiable to offer exemptions to people who object to general laws for reasons of religious conviction, secular conscience or personal integrity. In particular, there are good reasons to exempt people from general laws when objectors have reasons to object, when imposing the law on objectors would subject them to unique burdens and when exemptions policies do not impose costs on third parties’ (Navin and Largent, 2017).

Along the same lines, Navin and Redinger have recently argued that ‘[i]f the only available COVID-19 vaccine were one connected to abortion, then COVID-19 vaccine mandates would significantly constrain the religious liberty of people who object to vaccines for religious reasons’—which would also violate a legal requirement (in the USA) to ‘use the least restrictive means to promote compelling government interests whenever the state’s activity impedes religious liberty’ (Navin and Redinger, 2020). Similar legal constraints are in place in other countries, such as Canada. Thus, according to Navin and Redinger, mandating a vaccine without conscience exemptions, when it is possible to fund an alternative vaccine that would not compromise religious liberty, might be illegal in the USA. As we shall see, if funding alternative vaccines means diverting resources from more promising lines of research, thus delaying vaccine development, then funding an alternative vaccine is not the least restrictive means to promote government interests. A government’s primary interest is to have an effective vaccine *as soon as possible* (and as reasonable ethical and scientific standards allow).

The reasons offered by Navin and Redinger, and hinted at in the letters mentioned above, are not only ethical or legal in nature. There are also practical reasons: a COVID-19 vaccine linked to abortion might increase the rate of vaccine refusal. All in all, both ethical and pragmatic considerations translate into two claims. First, that we should not prioritize COVID-19 vaccine research linked to abortion. Second, that, if we do it and the first approved vaccine will be one linked to abortion, people with moral or religious opposition to it should be permitted to opt out in the name of freedom of religion and/or freedom of conscience.

It is important to point out that none of these religious authorities is against the ethical obligation to get vaccinated *per se*. All of them emphasize how important vaccines are for the protection of the public good and the most vulnerable members of society—which is why some think the Catholic social teaching implies a moral duty to vaccinate (Carson and Flood, 2017). The emphasis is rather on the request to divert resources into funding research on vaccines not linked to abortion, and when this is not done, on leaving people free to follow their conscience: vaccines in themselves are a good thing, but a moral agent’s opposition to abortion weighs more heavily, they claim.

In this article, we argue that respect for religious views and freedom should not hinder vaccine development and uptake—including the implementation of effective vaccination policies in a pandemic. This means that research on vaccines linked to abortion should be prioritized if and when that maximizes the chances of having a new vaccine sooner rather than later, which at the time of writing is the case with COVID-19 vaccine research. It also means that *if* mandatory vaccination will turn out to be necessary and ethically justified (an issue we are not going to take a stance on here), cost-free exemptions based on personal moral or religious views should not be granted. We agree that more religiously acceptable alternatives should be pursued, *other things being equal*. However, they should not be pursued at the cost of human lives.

We will first address the issues around research ethics. We will argue that even accepting (for the sake of argument) the premise that abortion is impermissible, it is not morally impermissible *to conduct research* on vaccines linked to abortion. Indeed, we will argue that such research is a moral imperative if there is a realistic prospect of it resulting in more safe and effective vaccines more quickly than with alternative lines of research.

We will then move on to discuss vaccination policies. We will argue that *if* at some point vaccination mandates (for instance in the form of vaccine or immunity passports) turn out to be ethically justified and necessary to achieve adequate vaccination coverage, there is no ethical justification for conscience or religious exemptions to vaccination mandates. Contrary to what defenders of conscience exemptions imply, it is likely that exemption policies will impose costs on innocent third parties. There are also other ethical requirements that need to be fulfilled apart from not imposing costs or harms to others—most notably fairness in vaccination policies.

Finally, we will briefly analyse claims about individual morality: we will argue that it can be morally permissible—even for people who consider abortion immoral or a ‘grave moral sin’—*to use* vaccines linked to abortions. Establishing this point as a matter of individual morality is important for two reasons. First, it could convince some to avoid claiming conscience exemptions. Second, one condition set out by Navin and Largent for the justification of conscience exemptions is, as per quote above, that ‘objectors have reasons to object’ (which we take to mean that they have reasons that meet some minimum threshold of validity, including that they are consistent with one’s own ethical principles). If it turns out that objectors do not have such reasons, then their argument for conscience exemptions does not apply.

These considerations apply to the vaccines currently approved and to the vaccines that will be researched and may be approved in the future as well.

## Research Ethics: Prioritizing Religious Freedom over Potentially Life-Saving Research is Unethical

Some people believe that abortion is wrong and that it is wrong to benefit from a serious wrongdoing like abortion. Is this a good enough reason to deprioritize research linked to practices perceived to be unethical in this way?

The proposition here can be read in two ways.

First, given a fixed research budget, at least some of the funds should be directed towards what people opposed to abortion take to be ethical research. But this would *de facto* mean reducing funding for research linked to abortion. If research linked to abortion is very promising—for example, there are vaccine candidates linked to abortion at an already advanced stage of study—this strategy would seriously risk delaying the moment when we have an effective vaccine, thus ultimately resulting in more avoidable deaths. It is worth keeping in mind that even if alternative vaccines are being developed and are very promising, we would still need more vaccines in the pipeline in order to guarantee enough supply at least in the short term. The criterion for allocating research funding to different lines of research should be how likely they are to be successful, not whether they are or are not linked to abortion. The choice to use human foetal cell lines for vaccine research is explained by the fact that such research represents a distinct and promising avenue to quickly deliver the required vaccine safely.

In addition to the considerations above, non-human cells are more likely to carry bacteria or viruses that could contaminate the resulting vaccine. For example, some polio vaccines developed in the 20th Century using cell lines derived from monkeys were found to contain a monkey virus that only by luck did turn out to be not harmful for humans (CPP (College of Physicians of Philadelphia) 2018). For all these reasons, it is not uncommon for vaccine research and manufacturing to be linked to human foetal cell lines. Current vaccines against rubella, chickenpox, hepatitis A and shingles were developed using cell-lines obtained from aborted foetuses. A non-human cell line might not produce safe and effective vaccines immediately, though it could do over the years. In the current pandemic, years is a length of time we cannot afford if we want to preserve human lives. Thus, funding alternative research just because it is not linked to abortion risks diverting resources into research that is less likely to be effective in the short term, and that would very likely cost lives.

Second, the request might be taken to mean that more funds should be put into vaccine research and development, and this additional funding should be reserved for research that is not linked to abortions. Of course, it is desirable to fund different streams of research in order to maximize the chances that at least some of those will deliver a vaccine soon. But the criterion for increasing and allocating research funding should be the maximization of the chances of having the safest and most effective vaccine sooner rather than later. Increasing research funding in order to satisfy some other criterion would be a missed opportunity to accelerate vaccine research and development and so to prevent avoidable deaths. Given the gravity of the current situation, and the fact that large numbers of lives are at stake, this is equivalent to slowing down such research by diverting currently allocated research budget to less promising lines of research.

In secular societies, what makes research ethical or unethical should be established on grounds of public reason, that is, on principles that can be justified without appealing to particular sectarian religious views—although one has good reasons to try to satisfy the preferences of everyone, including those with religious beliefs, when it comes at little or no significant cost. Ethical principles regulating medical research—especially research benefitting the whole population—cannot be *merely* religious in nature. Ethics typically sets boundaries to what researchers can permissibly do, which often justifiably slows down research. Unethical research would often mean quicker research. Since, under current circumstances, time means life, it is important that the ethical reasons for such boundaries be reasons that people with diverse religious views and people with no religion alike can share—given that their lives and their health are at stake, too. In other words, they need to be based on public reason, which ‘requires that our moral or political principles be justifiable to, or reasonably acceptable to, all those persons to whom the principles are meant to apply’ (Quong, 2017).

As for non-religious arguments for the moral impermissibility of abortion (e.g. those based on the potential of a foetus to become a person, or on the future a foetus is being deprived of), these are the subject of reasonable disagreement. In any case, current codes of ethics, both with regard to research and to clinical practice, do consider at least some instances of abortion ethically permissible (e.g. AMA, 2016), and they allow for data or tissues resulting from abortion to be used for research purposes with the of the woman who had the abortion (AMA, 2016, Opinion 7.3.4)). Moral uncertainty and moral disagreement about abortion do not make research linked to abortion unethical by current research ethics standards.

There are other possible ethical objections to using foetal cell lines in vaccine research. Although we do not have precise information about the foetus from which the tissues were harvested, it seems that the foetus, aborted in 1972 in the Netherlands, was ‘of unknown family history’ (US FDA (Food and Drug Administration) 2001). It was not too uncommon at that time to use foetal material from abortion without the consent of the woman (Wadman, 2018).

So, opponents might claim, this research is doubly wrong: women did not consent and it uses tissue obtained by immoral killing of the foetus. Even if the latter aspect might be the subject of reasonable disagreement, there likely is more widespread agreement on the wrongness of the former—or at least the wrongness of the former can more easily be established on the basis of public reason.

However, even though by today’s standards that foetal tissue procurement was unethical, it remains to be established whether the causal connection with previous unethical practices makes *current* research unethical. According to current standards and codes of research ethics, such causal link does not suffice to make current research unethical.

Where the benefits of current research are sufficiently valuable, benefitting from unethical past research is ethically acceptable—and we would argue, ethically required. Let’s grant for the sake of argument that abortion is morally wrong and/or that deriving cell lines from aborted foetuses for research purposes was unethical, for example because women did not give valid consent. Now let’s compare this with infamous Nazi experiments unanimously considered immoral. In the ‘immersion hypothermia project’, for instance, Nazi scientists immersed non-consenting concentration camp prisoners in tanks of icy water until they died. These experiments were grotesquely unethical. The purpose was to research the most effective treatment for German victims of immersion hypothermia, for instance fighter pilots shot down over the English Channel (Berger, 1990). Some data resulting from this research have been used and cited in subsequent research and dozens of scientific papers (Moe, 1984).

We agree that such data ought to be handled with care to avoid the risk of ‘being contaminated by the moral taint’ (Higgins *et al*., 2020)—for example by acknowledging the harm caused to the victims of such experiments whenever unethical research is cited (Moe, 1984, Higgins *et al*., 2020). However, current research guidelines do permit the use of such data for research purposes, including their reference in scientific papers, if significant benefit can result from such research; if the unethical nature of the original research is properly acknowledged; and if compelling ethical reasons can be provided for its use. For example, Opinion 7.2.2. of the Code of Ethics of the American Medical Association (AMA, 2016) states, with regard to past unethical experiments such as Nazi experiments during World War II or the US Public Health Service Tuskegee Syphilis Study, that

‘[i[n the rare instances when ethically tainted data have been validated by rigorous scientific analysis, are the only data of such nature available, and human lives would certainly be lost without the knowledge obtained from the data, it may be permissible to use or publish findings from unethical experiments. Physicians who engage with data from unethical experiments as authors, peer reviewers, or editors of medical publications should:

1. Disclose that the data derive from studies that do not meet contemporary standards for the ethical conduct of research.
2. Clearly describe and acknowledge the unethical nature of the experiment(s) from which the data are derived.
3. Provide ethically compelling reasons for which the data are being released or cited, such as the need to save human lives when no other relevant data are available.
4. Pay respect to those who were the victims of the unethical experimentation’

Baruch Cohen (1990) has noted that one way to defend the use of such data would be to see the use as a way of honouring the victims of such research. Their—involuntary—sacrifices could at least be seen to contribute to the good of humanity.

Understandably, this is not an interpretation that was shared by every holocaust survivor. However, as Stephen Post (1991) notes, some holocaust survivors held this view, provided there was a ‘clear and significant benefit’ to humanity.

One could argue that a similar line of reasoning could be applied to foetal material derived from aborted foetuses by those who think that abortions constitute a serious moral wrong. Refusing to use the results of such research could well be seen as showing disrespect to the aborted foetuses whose genetic material was used to undertake research the results of which are offering a clear and significant benefit to humanity.

Because time means life, if research on COVID-19 would be slowed down by the requirement not to be linked to abortion, there are indeed ethically compelling reasons for conducting such research even assuming that obtaining the cell lines in the first place was unethical. The burden of providing an argument to the contrary is on those who claim that current research ‘tainted’ by that causal link is unethical.

As a way of analogy, consider the case of research involving the destruction of human embryos for the purpose of embryonic stem cell procurement. After the discovery that induced pluripotent stem cells could be derived from human skin, there was some call for the abolition of research involving human embryonic stem cells. However, although pluripotent stem cells derived from adult cells have great potential, it is uncertain whether they can actually replace embryonic stem cells for all research and therapeutic purposes. If there is at least some reasonable chance of benefit in the continuation of research using embryonic stem cells, then this research ought to continue: there are no ethical reasons against it that can be established on the basis of public reason, and lives could be saved thanks to such research. Research that is not unethical on the basis of careful, rational assessment on secular grounds should only be stopped or prevented because of certain people’s or groups’ moral opposition if it is futile, that is, if there is no actual benefit that can plausibly be derived from it and that would outweigh people’s opposition to it. But, under the current circumstances, neither embryo research nor COVID-19 vaccine research linked to abortion are futile.

After all, chasing a regenerative medical cure or a vaccine for COVID-19 is like a horse race. We should let all horses run because we should not scratch a potential winner. Because time is lives, all plausible candidates must be tested on the basis of their chances to deliver the desired outcome sooner rather than later, unless there are convergent ethical reasons to eliminate a candidate (Devolder and Savulescu, 2006).

## Vaccination Policy: Prioritizing Religious Freedom over Public Health is Unethical

Another claim being made in the name of religious freedom and freedom of conscience is that, if COVID-19 vaccination policies are mandatory, there should be conscience exemptions for those who object to the vaccine because of its connection to abortion.

Now, whether COVID-19 vaccination should be made mandatory, and in that case for which people or groups, is an issue that would require a separate discussion. We want future COVID-19 vaccination policies to be maximally effective at preventing the largest numbers of lives lost prematurely and to strike the right balance between pursuing the collective interest and minimizing the risks for individuals, while exerting the lowest degree of coercion possible. Whether this will require mandatory vaccination will depend on a number of factors (safety profile of the vaccine, effectiveness in different groups, level of protection conferred, vaccine availability) which are unknown at the moment. However, *if* it turns out that mandatory vaccination will at some point be necessary or ethically justified, then there should be no conscience exemptions to mandates, whether for moral or religious reasons. Indeed, an ethical assessment of (i) whether future COVID-19 vaccination policies should be mandatory and (ii) for whom they should be mandatory should not be based on some people’s moral or religious opposition to the vaccine or on a principle of freedom of conscience and of religion.^4^

Before we argue why there should no exemption for conscientious objection, we should clarify what we mean by ‘no exemption’. What we mean is no exemption to the cost imposed for not being vaccinated. We do not mean that people must be forcibly vaccinated. Mandatory vaccination already exists with costs including fines (Italy), refusal of admission to school (USA), withdrawal of childcare benefits or child care (Australia). What we mean is that if you object, for whatever reason, you should pay the relevant cost, e.g. fine, withdrawal of childcare benefit, etc. What constitutes an appropriate form of coercion (or cost) is a separate issue and it should be proportionate (Savulescu, 2020).

Why should we not exempt conscientious objectors to vaccination? The comparison we made above with embryonic stem cell research is once again useful. Vaccination is different from regenerative medicine based on embryonic stem cells in an important respect. In the case of regenerative medicine, a patient refusing to use treatments based on embryonic stem cell research does not harm anyone else directly and, more generally, there is no significant public good at stake. In a public health system, there might be a case for requiring an individual to cover the costs for the healthcare system of them refusing a more effective treatment (Savulescu, 1998), but there is no compelling reason for overriding their autonomous decision about refusing certain treatments on personal moral grounds. However, in the case of vaccination, refusal to vaccinate both risks one’s own health and the health of others directly, and it represents a failure to contribute to an important public good (herd immunity against serious infectious diseases). Because each person is potentially a lethal threat to others, there is a requirement to ‘lay down your guns’ and reduce your threat to others (Flanigan, 2014). There is no valid religious duty or right to harm or to pose a serious risk of harm to innocent other people. Just as terrorists should not kill innocent people in the name of God, conscientious objectors should not pass on lethal viruses that could kill vulnerable people in the name of God.

Because the reasons not to allow conscientious exemptions to vaccination are based on considerations of harm prevention (both directly, by not exposing others to the virus, and indirectly, by contributing to herd immunity), the only valid alternative to vaccination would be extreme self-isolation. In this way, one would literally present no threat to others and would contribute to the collective effort of eradicating or containing the disease. However, religious leaders, and more generally those who claim a right to conscientious objection in the context of healthcare and public health, expect exemptions to be cost-free, for example by claiming that objectors should continue to receive state benefits and welfare. For example, Australian Catholic Archbishop Antony Fisher complained that ‘if the COVID-19 vaccine is linked to “no jab, no pay” rules, Catholic families could lose access to family payments if they refuse to vaccinate their children’.^5^

It is also important to recognize that rigorous self-isolation may reduce the direct threat to others, but it cannot contribute to herd immunity or to mitigating the wider harms of pandemic measures such as lockdowns. Indeed, by delaying herd immunity, it increases the likelihood of such harms occurring.

Conscientious objectors could say that there is an obvious difference between conscientious objection to vaccination and terrorism: terrorists will almost certainly kill innocent people; objectors are only imposing a very small risk of death on any individual. In a way, the argument goes, we impose small risks on others every time we sneeze or drive a car, and failure to be vaccinated is not much worse than these behaviours.

However, this response fails.

First, the risk posed by those who object to vaccination is not just that of infecting some other individual. Such infected individuals could go on and infect others (especially with diseases like COVID-19, where infected people are often asymptomatic and therefore not aware of being spreaders). The risk is therefore not as small as it might initially seem.

Second, we do take reasonable measures—that is, measures that are not too costly for an individual—to minimize risks on others posed by the aforementioned behaviours. We do have speed limits and other road rules, and the social norm of covering our mouth with our elbow when sneezing is now quite widespread. During this pandemic, we are often required to use face covering. Assuming vaccines are safe for any targeted group or group for which they are approved, vaccination is just a similarly reasonable and low-cost preventive measure. And we do impose costs when those rules are broken, such as speeding fines.

When we talk about harm prevention through vaccination, we are talking about responsibility that is not only individual, but also collective. Protecting people from infectious diseases requires collective effort—for instance, realizing herd immunity requires a large portion of the population to be vaccinated. The harm or risk of harm imposed on vulnerable people by absence of herd immunity—when herd immunity could be achieved through vaccination—is collectively produced.

Now, collective responsibility is a complex notion, both conceptually (Giubilini and Levy, 2018) and ethically. One ethical problem is that often individual contributions to the fulfilment of collective responsibilities and prevention of collective harm ‘does not make a difference’. Derek Parfit used now famous thought experiments to illustrate the problem. In one of these, for instance, a large number of people could contribute a pint of water to a collective cart that can be brought to the desert, where the water can be distributed among an equally large number of thirsty people. In the ‘harmless torturer’ case, a large number of people give each a very mild electric shock to a person; the shock from each individual torturer is negligible, but collectively the shocks cause severe pain to the victim (Parfit, 1984). In both cases, Parfit claims that individuals do have a moral obligation to contribute to harm prevention (either through action or inaction, respectively), even if the responsibility to prevent harm or to help out is collective and each individual would not ‘make a difference’.

Some of us have translated this principle into a principle of collective easy rescue in the case of vaccination, which grounds both a collective responsibility to realize herd immunity when the cost required to individuals is small, and an individual responsibility to contribute to that good by appealing to fairness in the distribution of the burdens (Giubilini *et al*., 2018, 2020). Thus, those who request not to be vaccinated on the basis of personal moral or religious reasons are in fact requesting to be exempted from making their fair contribution to collective harm prevention, and ultimately to an important global public good from which they will themselves benefit significantly. Controlling the spread of COVID-19 through a vaccine would protect everyone not only against the risks of COVID-19, but also against the economic damage, education costs, and risks for physical and mental health that pandemic management measures are creating. If we allow a principle of freedom of conscience to outweigh fairness in this context, we undermine one of the core principles regulating collective efforts and public goods in liberal societies. We should prioritize fairness over religious freedom, given that fairness has very significant intrinsic and instrumental value.

That fairness has intrinsic value is a point that would need a philosophical discussion to be established. For the purpose of the this article, suffice it to say that the point is *suggested* by the prominence fairness is given in many other public policies. Taxation policies are an obvious example: we want taxation policies to be not only effective at generating enough revenue to provide important goods, but also to be fair. In principle, we can achieve the former without the latter. And yet, we want taxation policies to be effective *and* fair.

But the value of fairness is not only intrinsic. It is also instrumental: we value fairness because fair policies are more likely to be successful by motivating people to comply. In other words, fairness would address the so-called ‘problem of assurance’: people are more motivated to contribute to public goods if they have enough reassurance that others would do the same . In the case of vaccination, fairness requires equal distribution of the burdens involved in the realization of herd immunity. It is not unfair to exempt people from requirements if these are too burdensome or risky on objective grounds—that is, grounds that can be assessed and reasonably established through public reason. If someone is at serious risk of significant side effects from vaccination (say, because of allergies), it is not unfair to exempt this person from vaccination requirements, because the sacrifice required of them to contribute to the public good would be too large.

Such risks can be assessed through public reason and on objective grounds (for instance, medical grounds). But claiming that personal moral or religious beliefs represent equally strong reasons for exemptions implies assuming a relativistic perspective, where religious views about abortion or complicity are as worthy of protection as harm prevention, fairness or the public good. Public policies in liberal democratic societies cannot be based on such ethical relativism (Blackford and Schuklenk, 2021). Preserving the public good, obligations towards other people (and most notably the obligation not to pose other people at easily preventable risk of harm), fairness requirements are all considerations that take priority over freedom of conscience when it comes to protecting people’s lives. Allowing conscience objectors to free-ride on others’ contributions to important public goods in the name of freedom of conscience would require giving freedom of religion or of conscience a moral weight that is difficult to justify (Leiter, 2014).

Defenders of religious exemptions might respond that those at risk can themselves be vaccinated, so that the conscientious objectors would not pose any risk to others. However, the problem is that not everyone can mount an effective immune response, not everyone can be vaccinated because of medical reasons, and immunity often wanes over time. If mandatory vaccination *were* warranted, it is an open question whether herd immunity could be achieved if personal or religious exemptions were allowed.

Some have argued that there is no point in imposing the costs or risks of vaccination on few people who would want to refuse the vaccine, when we can be confident that there is enough protection at the collective level. In that case, freeriding would not be a problem (Dawson, 2007). However, even accepting this point, we should not forget that religious or moral opposition to abortion is only one among the many possible and at least equally (un)reasonable moral beliefs one can appeal to in order to refuse vaccination: people might be opposed to the use of animals or animal products in research, might be committed to natural lifestyles, might refuse what they perceive to be authoritarian invasions of their bodily integrity, might be opposed to the capitalist system behind for-profit pharmaceutical companies, and so on. All of these beliefs, and many others, could be considered a matter of conscience in the same way as religious opposition to abortion.

Moreover, herd immunity is a very unstable condition and vaccination coverage is subject to constant change. Any measure that makes it more likely that vaccination uptake will drop creates risks for vulnerable members of the community—when vaccination uptake drops below herd immunity, it is too late and these people are already exposed to risks. We should prevent rather than remedy such situations. It is not acceptable to put people’s health and lives at significant risk because of convictions that cannot be defended by appeal to public reason.

A compromise that some of us have proposed is that of requiring those who request non-medical exemptions to provide alternative services that are roughly as burdensome and as valuable to society as the contribution they request the exemption from Giubilini *et al*. (2017). This would offer more reassurance that their request is sincere and not motivated by the intention to freeride. However, it is often difficult to establish what could make up for failure to vaccinate against infectious diseases. In this specific circumstance, where a pandemic is killing hundreds of thousands of people and paralyzing the world’s economy, it is hard to find suitable alternative contributions—except perhaps extreme self-isolation.

Respect for religious freedom or freedom of conscience more broadly can be part of what public reason requires, to the extent that it is a principle that all reasonable people—religious and non-religious alike—can accept. However, priorities need to be considered to determine what public reason requires in any specific context. What constitutes a reasonable compromise is not always what lies half-way between two opposite stances. Sometimes compromise itself is unreasonable, and the more extreme the circumstances, the more likely this is. In the case of public health policies in situations of emergency like the current pandemic, protecting people from COVID-19 is the priority even when it comes at *some* cost in terms of liberty. After all, we have accepted incredibly harmful restrictions of liberty through indiscriminate long lockdowns, quite regardless of whether the moral or political or religious beliefs of restricted individuals were consistent with accepting this kind of confinement. Consider, for example, the situation of a hard-core libertarian subject to lockdown restrictions. Whether lockdown has been unreasonable or effective is up for debate, of course, but the fact that most have accepted it suggests that in emergency situations preventing grave harm may legitimately take priority, and reasonable policies are those that prioritize preventing grave harm (whether or not this applies to lockdown).

Unfortunately, in conditions of scarce resources and when we have to run against time in order to preserve lives, it is reasonable to compromise a certain degree of freedom, including freedom of conscience and religious freedom, if that increases the chances of preserving more lives.

Of course, if we had a situation of herd immunity against COVID-19 or if those refusing vaccination were only the ones living isolated from the rest of society (as is the case for example for certain ultraorthodox groups traditionally opposed to vaccines), then there would be strong reasons to respect their freedom of choice with regard to vaccination, as their decisions would not affect others. However, we are not in that situation (yet).

In the same way, when we have ample availability of different vaccines, then the reasons for offering the choice of which vaccine on the basis of personal moral beliefs will be very strong (with an open question as to whether people choosing more expensive vaccines for reasons of conscience should be required to pay (part of) the additional cost—a question we are happy to leave open). However, again, we are far from that situation in most countries.

## Individual Morality and the Irrationality of Objection

Finally, it is interesting to explore on what grounds Catholics or other religious people object to vaccines linked to abortion in terms of their own doctrine. So far, we have assumed that the principle at stake is one of freedom to follow one’s religious views. However, religious doctrines that oppose abortion typically do not condemn *tout court* the use of vaccines linked to abortion. The argument from religious freedom might in this case rest on shaky foundations.

For example, the Roman Catholic social doctrine in general supports a moral duty to vaccinate in order to contribute to social goods and to prevent harm (Carson and Flood, 2017). Also, it supports the use of vaccines linked to abortion if they are necessary to protect vulnerable individuals and there is no suitable alternative.

As we mentioned above, also the official note of the Congregation for the Doctrine of Faith explicitly says that when alternative vaccines are not available, it is morally permissible for Catholics to use COVID-19 vaccines linked to abortion given the current grave risk the disease poses (Congregation for the Doctrine of Faith, 2020).

That document makes explicitly reference and relies on the views on the matter previously expressed by the Pontifical Academy for Life, which was established by the Pope specifically to study and promote the value of human life consistently with the Catholic doctrine. In 2017, the Academy published a short addendum (PAL, 2017) to one of their previous documents on vaccination (PAL, 2006). In this addendum, they state that using vaccines linked to abortion is in certain cases not only morally permissible but also morally mandatory. As they put it, ‘the moral obligation to guarantee the vaccination coverage necessary for the safety of others is no less urgent, especially the safety of more vulnerable subjects such as pregnant women and those affected by immunodeficiency who cannot be vaccinated against these diseases’ (PAL, 2017). Moreover, they ‘exclude that there is a morally relevant cooperation between those who use these vaccines today and the practice of voluntary abortion’ and they clarify that ‘all clinically recommended vaccinations can be used with a clear conscience and that the use of such vaccines does not signify some sort of cooperation with voluntary abortion’ (PAL, 2017).

Now this is not the only pronouncement by the Academy. It is an addendum to a longer original pronouncement. In the original document, the Academy did express a concern about cooperation in wrongdoing of people producing the vaccines, distributing them, using them, and also promoting their use (as in the case of many states imposing mandatory vaccinations). They had originally asserted that all these parties are complicit in wrongdoing, although to different degrees and in different ways. The degree of cooperation in wrongdoing of those using vaccines linked to abortion was judged to be very low, yet it required abstaining from the use of such vaccines unless they were necessary in order to protect the health of children and, indirectly, of the whole population.

According to the same original document, people using these vaccines were involved in a form of ‘very remote mediate [i.e. *indirect*] material cooperation, and thus very mild, in the performance of the original act of abortion’ (PAL, 2006). The remoteness of cooperation in the Catholic doctrine can refer to either or both a temporal and a physical relation to the abortion. In this case, for instance, remoteness consists in the fact that the abortions were performed about fifty years ago. Material cooperation denotes that an individual does not share and does not approve of the decision to perform the abortion, hence is not as complicit as someone who would encourage or approve of such act. The document also notes that this form of cooperation is passive. To say that the cooperation is passive, rather than active, indicates that the person using the vaccine only has a duty to denounce the fact that the vaccine was linked to an immoral act such as an abortion. Had they been actively complicit, they would have been in the position of doing something to prevent the abortion from happening.

In sum, the original document explained that the cooperation in wrongdoing of people using such vaccines was remote and not as morally problematic as that of people performing the abortion, or using the foetal cells for research. Yet, the document stated that, generally, one should refuse to be complicit to even such a remote degree, and therefore refuse to get vaccinated. Moreover, the document continued, they had a moral duty to ask for alternative vaccines developed without the use of aborted foetal tissues.

However, even in that older original document the Pontifical Academy for Life allowed for exceptions to the general prohibition. Passive indirect material cooperation does not require one not to cooperate *when there are good reasons to do so*. According to the same document, ‘it is right to abstain from using these vaccines if it can be done without causing children, and indirectly the population as a whole, to undergo significant risks to their health. However, if the latter are exposed to considerable dangers to their health, vaccines with moral problems pertaining to them may also be used on a temporary basis. The moral reason is that the duty to avoid passive material cooperation is not obligatory if there is grave inconvenience’ (PAL, 2006).

Given that the current pandemic context does pose serious health risks to many people, including vulnerable groups such as old people, it does not seem that religious freedom is a solid ground for requests of vaccine exemptions at least by Roman Catholics. Even assuming that religious freedom should be given significant moral weight in the formulation of vaccination policies, the principle does not seem to warrant a right at least for Catholics to be exempted from vaccination requirements *on the basis of their religion*.

What this demonstrates is that religious interpretation is flexible. Catholicism is only one denomination of Christianity. But what it shows is that religious leaders can reinterpret their prohibitions. We call on them to put life first, as many already proclaim to do.

## Conclusion

Even if one accepted the premise that abortion is unethical and/or that using aborted foetuses to derive cell lines for research purposes is unethical, using those cell lines now for research purposes is not unethical and it is actually ethically required.

Even assuming current research to develop a COVID-19 vaccine using cell lines from aborted foetuses is unethical, it is not unethical to benefit from such research by using this vaccine. Using the vaccine would not only protect those who are vaccinated but also protect other people around them and contribute to the public good of herd immunity and disease eradication, which will prevent a significant number of deaths.

It is important to stress that making vaccination mandatory does not imply that people would be forced to be vaccinated. Coercion can come in the form of withholding of benefits, fines or restriction of school entry. The costs of mandatory vaccination must be proportionate (Savulescu, 2021), but there should not be religious exceptions to incurring these costs.

Finally, general religious prohibitions are sometimes applied flexibly in specific cases, and COVID-19 vaccine research policy is one of those cases, given the large benefit that such vaccine can produce—we call on religious leaders to fully support and use the most effective, safe vaccine in COVID-19, and to support the most rapid means of developing it.

We should prioritize lives, fairness and responsibility over religious freedom. Delaying such research and vaccine uptake in the name of religious freedom is unethical because it risks causing more unnecessary deaths, and it is irresponsible because it stands in the way of fulfilling individual and collective responsibilities in containing potentially lethal infectious diseases. In the case of Roman Catholics at least, conscientious objection to any future COVID-19 vaccine linked to abortion is in an important sense irrational because it is inconsistent with the position of the most authoritative sources linked to the Catholic Church, and more generally with the moral imperative, which religious and non-religious people share, to protect the vulnerable and preserve lives.

## Funding

A.G. work was funded by the AHRC/UKRI (AH/V006819/1). J.S. work was funded in whole, or in part, by the Wellcome Trust [Grant number WT203132 and WT104848]. For the purpose of open access, the author has applied a CC BY public copyright licence to any Author Accepted Manuscript version arising from this submission. It also received support from the Australian Research Council: DP210102916 and DP190101547. Julian Savulescu, through his involvement with the Murdoch Children’s Research Institute, received funding through from the Victorian State Government through the Operational Infrastructure Support (OIS) Program.

## Conflict of Interest

 No conflict of interests.

## Footnotes

1 See e.g. the BBC report at https://www.bbc.co.uk/news/technology-54889084, last accessed 12 November 2020.

2 The letter is available at https://www.sciencemag.org/sites/default/files/Letter-to-FDA-urging-ethical-COVID-vaccines.April%2017%202020.pdf.

3 Archbishop Fisher OP: Let’s not create an ethical dilemma, *The Catholic Weekly*, 24 August 2020, at https://www.catholicweekly.com.au/archbishop-fisher-op-lets-not-create-an-ethical-dilemma/.

4 The issue we discuss here is only that of exemptions from vaccine mandates. We are not discussing here the issue whether people should be allowed the free choice of a vaccine over another once they decide to be vaccinated—some of us have addressed this issue elsewhere (Giubilini, Wilkinson, Savulescu, forthcoming).

5 Samantha Malden, COVID-19 vaccine: Archbishop Anthony Fisher condemns Oxford vaccine, *News.Com*, at https://www.news.com.au/world/coronavirus/australia/covid19-vaccine-archbishop-anthony-fisher-condemns-oxford-vaccine/news-story/7b94e83a5491b6a76964d9bddfcb495d.
